# Post-acute pre-discharge echocardiography in the long-term prognostic assessment of pulmonary thrombembolism

**DOI:** 10.1038/s41598-021-82038-1

**Published:** 2021-01-28

**Authors:** Nataša Kokalj, Matija Kozak, Borut Jug

**Affiliations:** Department of Vascular Diseases, University Medical Centre Ljubljana, Ljubljana, Slovenia

**Keywords:** Cardiology, Medical research

## Abstract

The aim of our study was to asses the long-term prognostic impact of post-acute, pre-discharge echocardiographic assessment of right ventricular (RV) dysfunction in patients with low- and intermediate-risk pulmonary embolism (PE). Consecutive patients with acute PE underwent post-acute, pre-discharge echocardiographic assessment of RV dysfunction (defined by: RV dilation, tricuspid anulus peak systolic excursion, or tricuspid regurgitation systolic velocity). A Cox multivariate survival mode was constructed to determine the prognostic impact of post-acute, pred-discharge RV dysfunction on all-cause mortality. 615 patients were included: 330 (54%) women, mean age 64 ± 18 years, 265 (43.1%) with post-acute, predischarge RV dysfunction. During follow-up (median 1068 days), 88 (14.3%) patients died. On Cox multivariate analyis, pre-discharge post-acute tricuspid regurgitation systolic velocity emerged as the only independent echocardiographic predictor of mortality (HR 1.73 for every 1 m/s increase; 95% confidence interval 1.033–2.897; *p* = 0.037). RV dysfunction persists in almost one half of PE patients in the post-acute phase on pre-discharge echocardiography; however, only tricuspid regurgitation systolic velocity independently predicts long-term prognosis.

## Background

Acute pulmonary embolism (PE) is a potentially fatal disease with mortality rates ranging from 5 to 36%^1–4^. Intermediate- and high-risk PE is associated with right ventricular (RV) dysfunction, which is present in up to 40% of normotensive and 70% of hypotensive patients, and can be assessed with transthoratic echocardiography^5^. There are several echocardiographic parameters of RV dysfunction, which have emerged as independent predictors of adverse outcomes. An echocardiographic right-to-left ventricular end diastolic diameter ratio of 0.9 or greater strongly predicts in-hospital mortality^6^. Impaired systolic RV function as determined by a tricuspid annular plane systolic excursion (TAPSE) below 16 mm is associated with a 2.1–2.6-fold increase in immediate post-discharge (30-day) mortality^7–9^. Tricuspid regurgitation severity, increased tricuspid regurgitation velocity (i.e. ≥ 2.7 m/s), and increased systolic pulmonary artery pressure (i.e. > 36 mmHg) have all been established as predictors of unfavourable short-term prognosis in patients with PE^8–10^.

Most of the present evidence focuses on the short-term prognostic role of acute RV hemodynamic changes in PE. Conversely, signs of RV dysfunction may persist in the post-acute phase, suggesting unfavourable PE resolution and possibly adverse long-term clinical consequences. However, the impact of persisting RV dysfunction on long-term prognosis in patients with PE remains uncertain. Thus, we wanted to assess the long-term prognostic impact of post-acute, pre-discharge echocardiographic signs of RV dysfunction in patients with PE.

## Methods

### Patients

This non-concurrent prospective study included 1070 consecutive patients with acute low- and intermediate-risk PE, admitted to the Department of Vascular Diseases of the University Medical Centre in Ljubljana, Slovenia, between January 2010 and September 2015. The study was carried out in accordance with relevant guidelines and regulations and informed consent was obtained for study participation. Ethical approval to report this study was obtained from the Republic of Slovenia National Medical Ethics Committee.

»Low-risk« pulmonary embolism was defined as pulmonary embolism with haemodynamic stability, without evidence of right ventricular dysfunction or damage to the heart muscle (normal values of troponin); »intermediate-low risk« pulmonary embolism was defined as pulmonary embolism with haemodynamic stability, with markers of right ventricular dysfunction *or* positive myocardial injury markers (elevated troponin levels); »intermediate high risk« pulmonary embolism was defined as pulmonary embolism with hemodynamic stability, with markers of right ventricular dysfunction *and* positive myocardial injury markers (elevated troponin levels). Patients who presented with hypotension and haemodynamic instability were not included in our study.

Patients with confirmed diagnosis of PE who underwent post-acute pre-discharge transthoracic echocardiography to assess RV function were included. Exclusion criteria were (1) patients with PE, who were treated as outpatients, (2) PE during pregnancy and (3) patients who underwent only emergency point-of-care echocardiography. Comorbidities (cardiovascular diseases, namely coronary artery disease, peripheral arterial disease, history of cerebrovascular events, chronic pulmonary disease), active malignant diseases, clinical presentation and management of PE were recorded at the time of admission to hospital.

N-terminal pro-brain natriuretic peptide (NT-proBNP) and Troponin I (TnI) values were also determined, using chemiluminescent method, with normal ranges of 86–486 pg/mL and 0–0.10 µg/L, respectively.

### Echocardiographic analysis

Pre-discharge 2D transthoracic echocardiography was performed in the post-acute phase of PE prior to patients' discharge from hospital on the GE Vivid 7 Ultrasound Machine model with 4 MHz phase array transducer.

RV dysfunction was defined by the presence of at least one of the following criteria: (1) the presence of RV dilation (i.e., RVOT parasternal long axis diameter > 30 mm or parasternal short axis proximal diameter > 35 mm or right-to-left ventricular end-diastolic apical 4-chamber diameter (RV/LV) > 0.9); (2) right atrium enlargement (apical 4-chamber RA area ≥ 18 cm^2^); (3) TAPSE < 18 mm; *or* (4) tricuspid regurgitation systolic velocity ≥ 2.7 m/s.

Left ventricular ejection fraction was measured in apical 4-chamber view (normal values > 55%), interventricular septum thickness and left ventricle posterior wall thickness (normal values for both parameters 0.6–1.0 cm).

The estimation of CVP was observed through measuring the diameter of the inferior vena cava, and its percentage change diameter during inspiration.

Primary outcome of the study was death of included patients; follow-up for mortality was conducted through national vital status database.

### Statistical analysis

Baseline characteristics of patients are presented as mean (± standard deviation) for normally distributed, as median (interquartile range) for non-normally distributed continuous variables, and as frequency (percentage) for categorical variables. Differences between groups were compared using the Student t-test for normally distributed continuous variables, Mann–Whitney *U* test for non-normally distributed continuous variables and Chi-square test to compare categorical variables. Kaplan–Meier curves and log-rank tests were used to evaluate event-free survival, *p* value < 0.05 was established as statistically significant. The impacts of echocardiographic parameters on survival were evaluated using Cox proportional hazard models, and were expressed as hazard ratio with corresponding 95% confidence intervals. A 2-tailed *p* value < 0.05 was established as the level of statistical significance for all tests. To measure inter-observer and intra-observer variability we calculated the Cronbach's Alpha coefficient of reliability for each one of the assessed echocardiographic parameters . For statistical analysis the program SPSS Statistics 23.0.0.2 (International Business Machines Corp., New York) was used.

The study was carried out in accordance with relevant guidlines and regulations and informed consent was obtained for study participation. Ethical approval to report this study was obtained from the Republic of Slovenia National Medical Ethics Committee.

## Results

### Baseline patient characteristics and clinical course of PE

**Figure 1:**
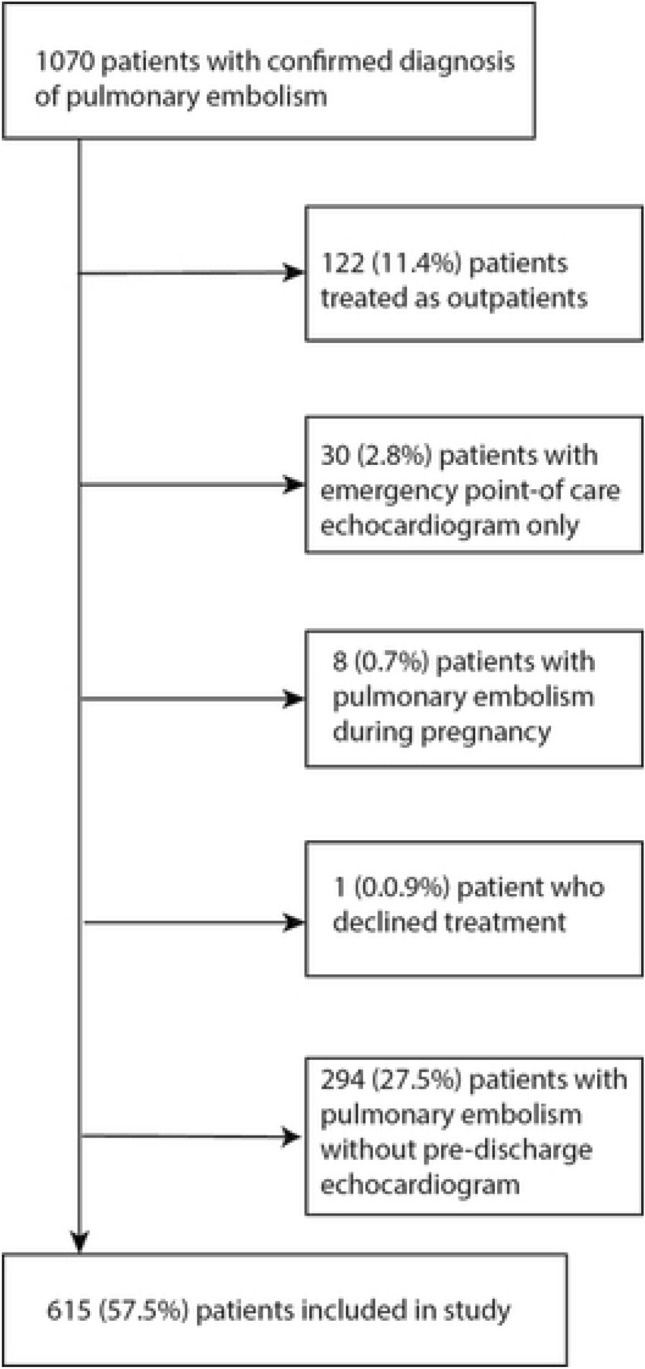
Patient flowchart.

Out of 1,070 patients who were admitted with the diagnosis of acute PE between January 2010 and September 2015, 615 patients met the inclusion criteria and had no exclusion criteria (Fig. 1).

Mean age was 64 ± 18 years; 330 (54%) patients were female. Patients with »high-risk« PE were exluded from our study, 154 patients were classified as»intermediate high risk«, 342 as »intermediate low risk«, and 119 patients as »low-risk« PE.

In 301 (49%) patients PE was considered unprovoked. During a median follow up period of 1.068 days, 88 (14.3%) patients died. 10 (1.6%) patients died during hospitalization. Patients who died were on average older, experienced longer hospital stays, had lower systolic blood pressure and higher heart rate at rest, more often impaired arterial oxygen saturation, higher NT-proBNP and troponin levels, and a significantly higher prevalence of cardiovascular co-morbidities and malignancies (Table 1).

**Table 1:** Baseline patient characteristics.

	All patients (n = 615)	Event-free (n = 527)	Event (n = 88)	*p* value
Age (years, mean, SD)	64 (18)	62 (18)	77 (10)	< 0.001
Sex (male, %)	285 (46.0)	245 (46.5)	40 (45.5)	0.857
SBP (mmHg, median, IQR)	134 (120–150)	135 (122–152)	129 (98–145)	0.016
Hospital stay (days, SD)	12 (11)	11 (7)	20 (17)	< 0.001
Cardiovascular disease (%)	92 (15.0)	67 (12.7)	25 (28.4)	< 0.001
Chronic heart failure (%)	37 (6.0)	23 (4.4)	14 (15.9)	< 0.001
Chronic pulmonary disease (%)	55 (8.9 )	46 (8.7 )	9 (10.2 )	0.648
Malignancy (%)	104 (16.9 )	71 (13.5 )	33 (37.5 )	< 0.001
Heart rate (bpm, median, IQR)	90 (76–104)	89 (75–103)	98 (81–101)	0.002
eGFR (ml/min, median, IQR)	72 (57–89)	75 (57–90)	58 (50–71)	< 0.001
NT- proBNP (pg/mL, median, IQR)	1113 (205–3518)	1038 (186–2961)	2654 (944–6463)	0.011
Arterial oxygen saturation (%, SD)	93 (5)	94 (4)	91 (7)	0.001
TnI (µg/L, IQR)	0.03 (0.01–0.24)	0.03 (0.01–0.24)	0.10 (0.02–0.32)	< 0.001

Abbreviations: eGFR—estimated glomerular filtration rate, IQR—interquartile range, NT-proBNP—N-terminal pro-brain natriuretic peptide, SBP—systolic blood pressure, SD—standard deviation, TnI—troponin I.

### Anticoagulant treatment

At admission, 12 (1.9%) patients received thrombolysis, 176 (28.6%) patients were treated with unfractionated heparin, and 427 (69.5%) patients with low-molecular-weight heparin.

At discharge, 325 (52.8%) received vitamin K antagonists, 182 (29.6%) patients received non-vitamin K-dependent direct oral anticoagulant agents, and 108 (17.6%) patients were discharged with low-molecular-weight heparin.

### Echocardiographic evaluation

Echocardiography as a mandatory pre-discharge diagnostic procedure was introduced in the clinical pathway for PE at the institution in 2008. Despite adherence to pre-discharge echocardiography was incomplete, only 27% of patients were discharged without a pre-discharge echocardiographic appraisal. Median time from PE diagnosis to echocardiography was 10 days.

Signs of RV dysfunction—either RV dilation, TAPSE < 18 mm or tricuspid regurgitation systolic velocity ≥ 2.7 m/s—on post-acute pre-discharge echocardiography were appreciated in 265 (43.1%) patients. The prevalence of RV dysfunction was significantly higher in patients who went on to experience an event (53.4 vs. 41.3%; *p* = 0.024). Patients who died also had statistically significant lower left ventricular ejection fraction (LVEF) and higher left ventricular wall thickness (Table 2).

**Table 2:** Echocardiographic parameters in patients with pulmonary embolism.

	All patients (n = 615)	Event-free (n = 527)	Event (n = 88)	*p* value
Right ventricular dysfunction* (%)	265 (43.1)	218 (41.3)	47 (53.4)	0.024
Right ventricular dilation (%)	241 (39.2)	196 (37.2)	43 (48.8)	0.038
Right atrium enlargement (%)	98 (15.9)	77 (14.6)	21 (23.8)	0.028
TAPSE (mm, SD)	23 (3)	21 (5)	18 (6)	0.009
Tricuspid regurgitation systolic velocity, (m/s, SD)	2.9 (1.7)	2.8 (1.6)	3.1 (1.8)	< 0.001
RVSP (mmHg, SD)	33 (11)	32 (11)	39 (13)	< 0.001
LVEF, % (SD)	63.8 (8.7)	63.9 (8.6)	61.0 (11.3)	0.017
IVS thickness > 1.1 cm (%)	211 (34.3)	180 (34.2)	31 (35.2)	0.046
LVPW thickness > 1.1 cm (%)	196 (31.9)	167 (31.2)	29 (33.0)	0.037

*Any of the following: RV dilation, TAPSE < 18 mm or tricuspid regurgitation systolic velocity ≥ 2.7 m/s.

Abbreviations: IVS—interventricular septum, LVEF—left ventricular ejection fraction, LVPW—left ventricular posterior wall, RVSP—right ventricular systolic pressure, SD—standard deviation, TAPSE—tricuspid annular plane systolic excursion.

A high degree of reliability was found between measurements of echocardiographic parameters**.** The Intraclass Correlation Coefficient (ICC) values were 0.99 for assessing right ventricular dilation, 0.88 for right atrium enlargement, 0.98 for TAPSE, 0.98 for tricuspid regurgitation systolic velocity, 0.96 for left ventricular ejection fraction, 0.97 for assessing interventricular septum thickness and 0.97 for left ventricular posterior wall thickness.

On univariate analysis age, malignant etiology of PE, arterial oxygen saturation, systolic blood pressure, heart rate, estimated glomerular filtration rate, N-terminal pro-brain natriuretic peptide (NT-proBNP) levels, troponin levest, left ventricular ejection fraction (LVEF) and tricuspid regurgitation systolic velocity–but not gender, RV dilation or TAPSE—were significantly associated with all-cause long-term mortality.

After multivariate analysis adjustment, however, only NT-proBNP levels and tricuspid regurgitation systolic velocity emerged as an independent predictors for adverse outcomes—even after adjusting for age, gender, malignant etiology of PE, arterial oxygen saturation, systolic blood pressure, heart rate, estimated glomerular filtration rate, troponin levels, LVEF and NT-proBNP levels (Table 3).

**Table 3:** Cox proportional hazard uni- and multivariate predictors of long-term mortality.

	Univariate analysis			Multivariate analysis		
	*p* value	HR	95% CI	*p* value	HR	95% CI
Age	< 0.001	1.065	1.044–1.086	0.061	1.073	0.997–1.156
Gender	0.959	1.011	0.665–1.537	0.864	1.182	0.220–6.351
Malignant etiology of pulmonary embolism	< 0.001	3.155	2.047–4.862	0.133	3.321	0.695–15.877
Arterial oxygen saturation	< 0.001	0.943	0.919–0.969	0.057	0.970	0.942–1.000
Systolic blood pressure	0.032	1.010	0.981–0.999	0.801	0.995	0.959–1.003
Heart rate	0.001	1.019	1.008–1.030	0.560	1.015	0.988–1.096
eGFR	< 0.001	1.030	0.959–0.984	0.131	1.041	0.988–1.096
Log NT-proBNP	0.008	2.060	1.21–3.52	0.035	1.869	1.045–3.344
LVEF	< 0.001	0.961	0.942–0.982	0.231	0.982	0.954–1.012
TnI	< 0.001	1.000	1.000–1.000	0.507	0.973	0.899–1.054
Right ventricular dilation	0.113	1.403	0.923–2.130			
TAPSE	0.196	0.992	0.979–1.004			
Tricuspid regurgitation systolic velocity	< 0.001	2.402	1.555–3.720	0.037	1.730	1.033–2.897

Abbreviations: CI—confidence interval, HR—hazard ratio, eGFR—estimated glomerular filtration rate, LVEF—left ventricular ejection fraction, NT-proBNP—N-terminal pro-brain natriuretic peptide, TAPSE—tricuspid annular plane systolic excursion, TnI—troponin I.

**Figure 2:**
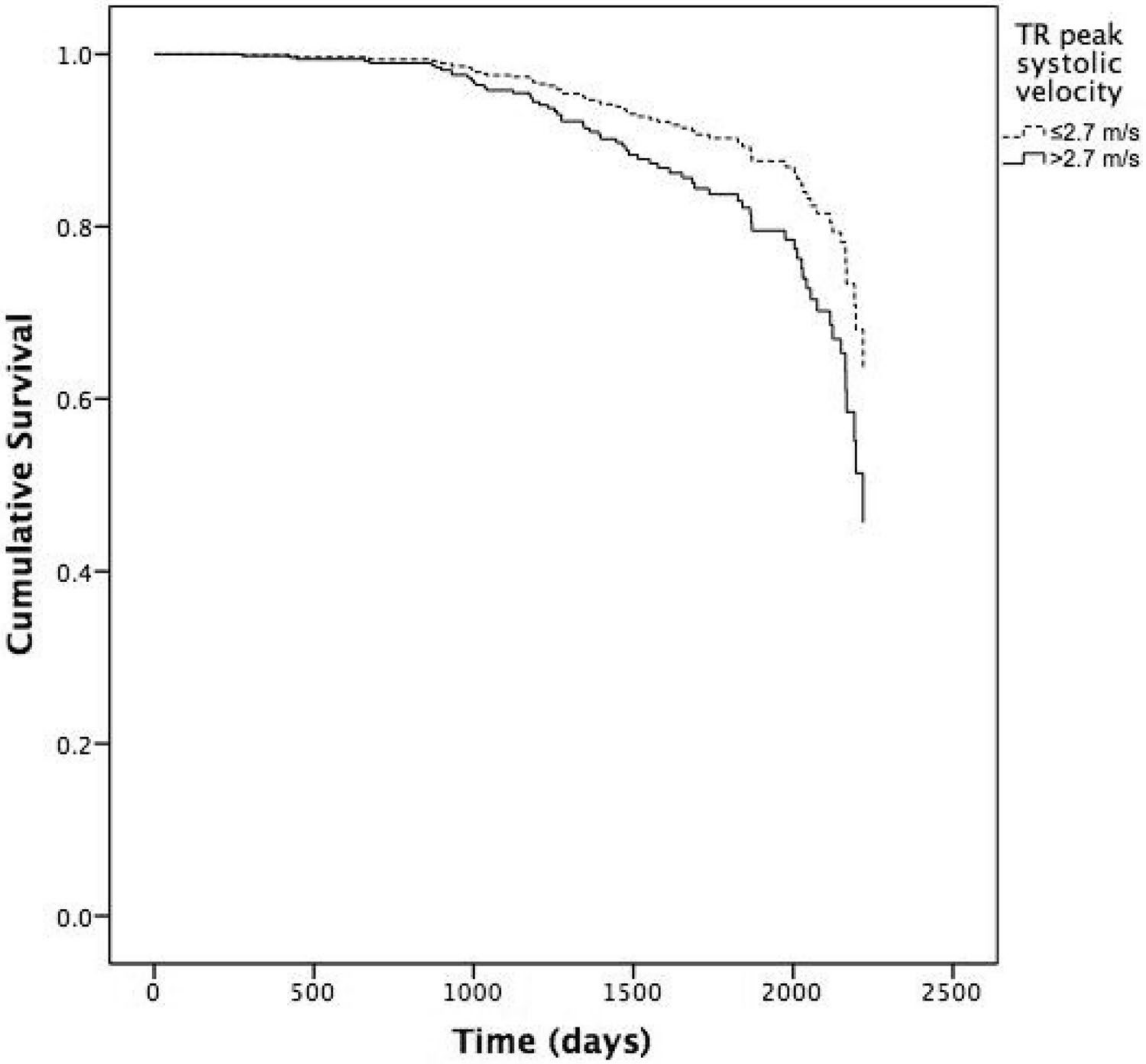
Survival curves and HR (adusted for age, gender, malignant etiology of pulmonary embolisn, arterial oxygen saturation, systolic blood pressure, heart rate, estimated glomerular filtration rate, troponin levels, N-terminal pro-brain natriuretic peptide levels and left ventricular ejejction fraction). Abbreviations: TR—tricuspid regurgitation.

A 1.73-fold increase in all-cause mortality was associated with every 1 m/s increase in tricuspid regurgitation systolic velocity (95% confidence interval 1.033–2.897; *p* = 0.037) (Fig. 2).

## Discussion

Post-acute pre-discharge echocardiographic signs of RV dysfunction are associated with unfavourable prognosis. In our study, almost one half of patients in the post-acute phase of PE had echocardiographic signs of residual RV dysfunction; more importantly, parameters suggesting residual RV dysfunction were strongly associated with long-term mortality. However, on multivariate analysis, only tricuspid regurgitation systolic velocity emerged as an independent predictor of prognosis after adjusting for age, gender, malignant etiology of PE, arterial oxygen saturation, systolic blood pressure, heart rate, estimated glomerular filtration rate, troponin levels, LVEF and NT-proBNP levels.

Previous studies have already shown that acute RV dysfunction, as appreciated with emergency setting echocardiographic assessment, predicts short-term prognosis^6,9–13^. In a study, conducted by Ciurzyński et al. a novel echocardiographic parameter was presented—The tricuspid regurgitation peak gradient (TRPG)/TAPSE, which could help identify patients with short-term increased risk of death or hemodynamic deterioration^14^.

Pathophysiology of PE, however, suggests that RV dilation and dysfunction may persist in the medium- and longer-term, and that resolution or persistence of post-acute RV dysfunction may predict long-term prognosis in this patient population^5,6,9–13,15^. Our results indeed suggest that prevalence of residual RV dysfunction remains high in pre-discharge patients with PE; as many as 46.8% of our patients met at least one criterion for RV dysfunction in the post-acute phase before discharge from hospital, which is consistent with reports of longer term post-acute RV dysfunction in patients who were considered candidates for thrombolysis because of intermediate-risk PE in the PEITHO trial^16,24^.

More importantly, our findings suggest that post-acute RV dysfunction is associated with poor long-term prognosis. Echohocardiographic signs of RV dysfunction were significantly more prevalent in patients who went on to experience an adverse event.

However, only tricuspid regurgitation systolic velocity emerged as an independent echocardiographic predictor of survival after multivariate adjustment. Our results confirm previous studies pointing to tricuspid regurgitation systolic velocity as an important prognostic predictor of adverse short-term outcomes in patients with PE^10^. The association between tricuspid regurgitation systolic velocity and survival is expected. On the one hand, tricuspid regurgitation systolic velocity indicates increased pulmonary pressure in patients with PE and may therefore suggests disease severity; on the other hand, tricuspid regurgitation may also reflect the presence of undiagnosed concomitant pulmonary disease or left ventricular impairment, which in itself represents an unfavorable prognostic factor in patients with PE^17–21^.

In our analysis, a late separation of Kaplan–Meyer curves implies a long-term prognostic impact of increased tricuspid regurgitation on mortality, and suggests that the presence of this echocardiographic finding may primarily represent a marker of ongoing underlying conditions (such as chronic pulmonary disease) rather than prognostically unfavorable hemodynamic derangements in PE. In fact, several studies demonstrated that chronic obstructive pulmonary disease, for one, is assocciated with pulmonary hypertension and higher tricuspid regurgitation velocity, and also increased risk for, and worse outcome of, PE^22,23^.

Identification of prognostic factors in patients with acute PE represents an important issue in the risk assessment of, and therapy guidance for, patients with PE. High prevalence and prognostic implications of RV dysfunction in the post-acute phase of PE suggest that RV hemodynamic derangements may persist in patients with PE and, more importantly, may be in turn associated with unfavourable long-term prognosis. Thus, echocardiographic appraisal of patients with PE in the post-acute, pre-discharge phase may provide guidance for intensity and length of their clinical follow-up.

In our study we have identified some limitations. Firstly, patients in our study were diagnosed with intermediate-risk PE, thus our findings cannot be extrapolated to other patient populations with PE (such as those in hemodynamic compromise or those with low-risk PE managed as outpatients). Secondly, ours was a single-centre study; although providing data from a major national referral centre catering one third of the country's population, limitations inherent to single-centre observations need to be accounted for. Thirdly, there are biases inherent in the non-concurrent observational design of our study–such as observational nature of findings and data collection issues, which need to be taken into account when interpreting our findings. While the echocardiographic protocol was the same throughout the study period (adherent to the PEITHO study RV appraisal criteria), 187 (30%) patients were missing at least one RV appraisal criterion. Missing data were handled statistically by the mean imputation method. To measure inter-observer and intra-observer variability we calculated the Intraclass corelation coefficient of reliability. Based on the results, the inter-observer and intra-observer reliability was good to excellent. Importantly, only patients with echocardiographic appraisal were included in the analysis. While post-acute, pre-discharge echocardiography in patients with PE is recommended in all patients at our institution, 27% did not undergo testing and this limits the generalisability of our data. The primary outcome of our study was all-cause mortality, this data was conducted through national vital status database, which holds data for all Slovenian residents; however the data on the causes of death could not be obtained, which is also one of the limitations of our study. In our study we obtained data on anticoagulant therapy which was applied in the in-hospital setting and management of anticoagulant treatment after the patients were dismissed from hospital was carried in the outpatient clinic; the data of patients' adherence to anticoagulant treatment was not obtained; this missing data, however, could have influence on the outcome and this is also one of the limitations of our study.

Regardless, ours is the first study to address the long-term prognostic impact of post-acute, pre-discharge echocardiography in PE. Our results have shown that the prevalence of persistent RV dysfunction is high and confers unfavourable long-term prognosis; especially tricuspid regurgitation systolic velocity emerged as an independent predictor of all-cause long-term mortality and should be preferentially accounted for in the long-term risk-stratification strategies of patients with PE.

## References

[1] Heit JA (2001). The epidemiology of venous thromboembolism in the community. Thromb. Haemost..

[2] Silverstein MD (1998). Trends in the incidence of deep vein thrombosis and pulmonary embolism: a 25-year population-based study. Arch. Intern. Med..

[3] Torbicki A (2008). Guidelines on the diagnosis and management of acute pulmonary embolism: the Task force for the diagnosis and management of acute pulmonary embolism of the European Society of Cardiology (ESC). Eur. Heart J..

[4] Chughtai HL, Janjua M, Matta F, Jaweesh F, Stein PD (2011). Predictors of in-hospital mortality in patients receiving thrombolytic therapy for pulmonary embolism. Clin. Appl. Thromb. Hemost..

[5] Ten Wolde M, Söhne M, Quak E, Gillavry MRM, Buller HR (2004). Prognostic value of echocardiographically assessed right ventricular dysfunction in patients with pulmonary embolism. Arch. Intern. Med..

[6] Frémont B (2008). Prognostic value of echocardiographic right/left ventricular end-diastolic diameter ratio in patients with acute pulmonary embolism: results from a monocenter registry of 1,416 patients. Chest.

[7] Tousignant C, Kim H, Papa F, Mazer CD (2012). Evaluation of TAPSE as a measure of right ventricular output. Can. J. Anaesth..

[8] Rudski LG (2010). Guidelines for the echocardiographic assessment of the right heart in adults: a report from the American Society of Echocardiography endorsed by the European Association of Echocardiography, a registered branch of the European Society of Cardiology, and the Canadian Society of Echocardiography. J. Am. Soc. Echocardiogr..

[9] Lobo JL (2014). Prognostic significance of tricuspid annular displacement in normotensive patients with acute symptomatic pulmonary embolism. J. Thromb. Haemost..

[10] Profitis K, Lu K, De Silva D (2011). Tricuspid regurgitation is an independent predictor of mortality in acute pulmonary embolism. Heart Lung Circ..

[11] Cho JH (2014). Prognostic implications of diastolic dysfunction in patients with acute pulmonary embolism. BMC Res. Notes.

[12] Kreit JW (2004). The impact of right ventricular dysfunction on the prognosis and therapy of normotensive patients with pulmonary embolism. Chest.

[13] Kucher N, Rossi E, De Rosa M, Goldhaber SZ (2005). Prognostic role of echocardiography among patients with acute pulmonary embolism and a systolic arterial pressure of 90 mm Hg or higher. Arch. Intern. Med..

[14] Ciurzyński M (2018). Tricuspid regurgitation peak gradient (TRPG)/Tricuspid annulus plane systolic excursion (TAPSE)—a novel parameter for stepwise echocardiographic risk stratification in normotensive patients with acute pulmonary embolism. Circ. J..

[15] Matthews JC, McLaughlin V (2008). Acute right ventricular failure in the setting of acute pulmonary embolism or chronic pulmonary hypertension: A detailed review of the pathophysiology, diagnosis, and management. Curr. Cardiol. Rev..

[16] Konstantinides SV (2017). Impact of thrombolytic therapy on the long-term outcome of intermediate-risk pulmonary embolism. J. Am. Coll. Cardiol..

[17] Dahhan T (2016). Clinical and echocardiographic predictors of mortality in acute pulmonary embolism. Cardiovasc. Ultrasound.

[18] Carson JL, Terrin ML, Duff A, Kelley MA (1996). Pulmonary embolism and mortality in patients with COPD. Chest.

[19] Koelling TM, Aaronson KD, Cody RJ, Bach DS, Armstrong WF (2002). Prognostic significance of mitral regurgitation and tricuspid regurgitation in patients with left ventricular systolic dysfunction. Am. Heart J..

[20] Hung J (1998). Usefulness of echocardiographic determined tricuspid regurgitation in predicting event-free survival in severe heart failure secondary to idiopathic-dilated cardiomyopathy or to ischemic cardiomyopathy. Am. J. Cardiol..

[21] Nath J, Foster E, Heidenreich PA (2004). Impact of tricuspid regurgitation on long-term survival. J. Am. Coll. Cardiol..

[22] Poalsen SH, Noer I, Muller JE, Frandsen JL (2001). Clinical outcome of patients with suspected pulmonary embolism. A follow-up study of 588 consecutive patients. J. Intern. Med..

[23] Bahloul M (2015). Incidence and impact outcome of pulmonary embolism in critically ill patients with severe exacerbation of chronic obstructive pulmonary diseases. Clin. Respir. J..

[24] Meyer G (2014). Fibrinolysis for patients with intermediate-risk pulmonary embolism. N. Engl. J. Med..

